# The Treatment of Marasmus, or Simple Infantile Atrophy

**Published:** 1903-04-25

**Authors:** Eric Pritchard


### THE TREATMENT OF MARASMUS, OR SIMPLE INFANTILE ATROPHY.

By Eric Pritchard, M.A., M.D. (Oxon), M.R.C.P. London.

Since the rational treatment of disease is based on a correct understanding of the pathological processes which underlie the condition it is hardly surprising that the treatment of infantile atrophy (athrepsia or marasmus) a disease which has no known pathology, is empirical in the extreme and as a rule very disappointing.

I do not propose to discuss the various theories which have been adduced in explanation of this pernicious form of atrophy : it will be sufficient for my purpose to recapitulate the chief views which are held on this question. These are :—(1) That infantile atrophy is due to a general starvation of the tissues as a consequence of, and secondary to, primary atrophy of the mucous membrane of the bowel; (2) that the condition is due to starvation of the tissues owing to want of proper digestion of the food; (3) that the condition is due to a general toxæmia produced by the decomposition of food in the alimentary tract. For my part I do not regard any of these theories as entirely satisfactory for various reasons which I do not propose to enter into now. I believe it not improbable that simple infantile atrophy is due to a general inability on the part of the tissues to assimilate the nutrient supplies which reach them by way of the blood, lymph stream or alimentary tract. That is to say, that the disease is a general one and not confined exclusively to the digestive system. I further think it possible that this inability of nutrition is partly due to hereditary or pre-natal causes, and partly to post-natal overstimulation by means of improper food. In other words, simple infantile atrophy may be regarded as representing a condition of nutritional fatigue wherein the tissue cells generally are incapable of responding to the food stimuli which reached them from within or from without. Whether this view is justifiable or not, experience has convinced me that a line of treatment based on this pathological hypothesis, answers exceedingly well in practice. Assuming that there is a general condition of fatigue of the processes of cell nutrition, the rational indications for treatment should be in principle as follows :—(1) To place the tissues under the most favourable circumstances for securing rest and to provide time for recovery; (2) to vary the form of food stimulus as much as and as frequently as possible. To carry out these general principles the following details in the treatment should be observed :—

1. To give the digestive tract and tissues generally as much rest as possible, the quantity of food must be reduced to an absolute minimum.

2. To stimulate nutrition the quality of the food must be varied in character and stimulating in quality.

3. To conserve energy and maintain the bodily temperature the patient should be wrapped in cotton-wool.

4. To ensure a free supply of oxygen he must be kept in a large well-ventilated room.

5. To maintain the nutrition of the muscles they should be massaged daily.

6. To ensure the removal of waste products the infant should be given plenty of water to drink.

In fact the fire of life must be supplied with fuel of the best quality and of the most combustible nature. The cinders and ashes must be well raked out and an adequate draught maintained. Under favourable conditions of this kind the smouldering embers of vitality may ultimately be fanned into an active and vigorous flame.

On a correct appreciation of the dietetic indications more than on anything else depends the successful issue of the treatment. Experience unquestionably proves that the sluggish and apathetic nutrition of marasmic infants will not respond to the feeble stimulus of an ordinary milk diet. Food which has more exciting and stimulating qualities is required. And further, I imagine it must be the experience of all who have treated many of these cases to find that although entire change in the diet may prove successful for a time nevertheless the effect soon passes off, and further changes have to be made. These results are in entire agreement with the conception of the pathology of marasmus which I have sketched above. To ring the changes in diet sufficiently frequently and to find daily new forms of stimuli it is necessary to have at hand a considerable *repertoire* of formulæ for the preparation of food, which must contain the essential elements for maintaining nutrition. We must have representatives of proteid, carbo-hydrate and fatty elements, and in addition salts and extractives which, though not to be regarded directly as foods, are nevertheless essential elements of any dietary.

The following representatives of the proteid class of foods may be regarded as affording sufficient variety: raw meat juice, Plasmon, Protene, Prideaux's casumen, somatose, invalid bovril, Vegox, beef-tea, mutton broth, veal jelly, Benger's peptone jelly. As regards carbohydrate elements many of the patent preparations are particularly useful; more especially for the reason that as a rule they contain in addition small quantities of proteids and fats. To mention a few :—Frame food, Mellin's food, Benger's food, Horlick's malted milk, and, among pure carbo-hydrates, honey and milk sugar. To find satisfactory varieties of assimilable fats is a more difficult matter. Cream itself is a most useful stand-by, but cream, even in infinitesimal quantities, seems to disagree in certain cases. Under such circumstances I have found Virol very useful. Lahmann's vegetable milk, which contains a considerable proportion of fat obtained from nuts, is also an excellent preparation. Emulsion of almond oil or cod-liver oil sometimes answers admirably, and dripping well rubbed up with some carbohydrate preparation, such as frame food or bread jelly, constitutes an excellent change. It is impossible in a short article to describe many of the possible combinations which may be derived from mixing together representatives of the foods described above. A little ingenuity and resource will, however, enable the reader to prepare any number of combinations by following the method adopted in preparing the following types :—

1. One teaspoonful of bread jelly or frame food, well rubbed up with 10 drops of cream (dripping, Lahmann's vegetable milk or emulsion of almond oil), two teaspoonfuls of raw meat juice, and water to make 1½ ounces.

2. One teaspoonful of bread jelly or frame food, one half teaspoonful of virol, one half ounce of strong beef tea, or 15 drops of invalid bovril, and water make 1½ ounces.

3. Prideaux's casumen half teaspoonful, Mellin's food half teaspoonful, virol half teaspoonful, and water to 1½ ounces.

4. Frame food one teaspoonful, cream 10 drops, or virol 15 drops, Benger's pepton jelly one teaspoonful, water to 1½ ounces.

If necessary a few drops of brandy may be added to all of these mixtures. For an infant of four months 1½ ounce of any of the above mixtures, every two and a-half or three hours, is at first amply sufficient. Sterilised water flavoured with a little Bovril or other stimulating preparation should be given freely between the regular times of feeding.

In conclusion, I would strongly advise any practitioner who has the management of one of these cases to secure the services of a trained nurse, to follow the effect of his treatment by weighing the infant carefully at least twice a week, and to keep a systematic record of the progress; to exercise the greatest patience, and to be content at first with any improvement however slow.

